# Efficacy of Er:YAG laser irradiation for decontamination and its effect on biocompatibility of different titanium surfaces

**DOI:** 10.1186/s12903-021-02006-z

**Published:** 2021-12-18

**Authors:** Peijun Huang, Xue Chen, Zhongren Chen, Min Chen, Jinzhi He, Lin Peng

**Affiliations:** 1 State Key Laboratory of Oral Diseases, National Clinical Research Center for Oral Diseases, West China Hospital of Stomatology, Sichuan University, Chengdu, China; 2 Chongqing Key Laboratory of Oral Diseases and Biomedical Science, Stomatological Hospital of Chongqing Medical University, Chongqing, China

**Keywords:** Er:YAG laser, Microbial biofilm, Peri-implantitis, Titanium surface, Biocompatibility

## Abstract

**Background:**

Erbium yttrium–aluminum–garnet (Er:YAG) laser have been shown to be suitable for decontamination of titanium surfaces at a wide range of energy settings, however, high intensity of laser irradiation destroy titanium surface and low intensity cannot remove enough microbial biofilm. The aim of this study was to investigate the optimal energy setting of Er:YAG laser for decontamination of sandblasted/acid-etched (SLA) and hydroxyapatite (HA) titanium surfaces.

**Material and methods:**

After supragingival biofilm construction in vivo, SLA and HA titanium discs were divided into three groups: blank control (BC, clean discs), experimental control (EC, contaminated discs) and experimental groups (EP, contaminated discs irradiated by Er:YAG laser at 40, 70, and 100 mJ/pulse). Scanning electron microscopy (SEM), live/dead bacterial fluorescent detection, and colony counting assay were used to detect the efficacy of laser decontamination. To investigate the effect of laser decontamination on titanium surface biocompatibility, MC3T3-E1 cell adhesion and proliferation activity were examined by SEM and CCK-8 assay.

**Results:**

Er:YAG laser irradiation at 100 mJ/pulse removed 84.1% of bacteria from SLA titanium surface; laser irradiation at 70 and 100 mJ/pulse removed 76.4% and 77.85% of bacteria from HA titanium surface respectively. Laser irradiation improved MC3T3-E1 cell adhesion on both titanium surfaces. For SLA titanium discs, 100 mJ/pulse group displayed excellent cellular proliferation activity higher than that in BC group (*P* < 0.01). For HA titanium discs, 70 mJ/pulse group showed the highest activity comparable to BC group (*P* > 0.05).

**Conclusions:**

With regards to efficient microbial biofilm decontamination and biocompatibility maintenance, Er:YAG laser at 100 mJ/pulse and 70 mJ/pulse are considered as the optimal energy settings for SLA titanium and HA titanium surface respectively. This study provides theoretical basis for the clinical application of Er:YAG laser in the treatment of peri-implantitis.

## Background

Peri-implantitis is one of the most frequent complications of implant therapy; it has been associated with inflammation in the peri-implant mucosa and progressive loss of supporting bone, eventually resulting in the loss of the functioning osseointegrated implants [1]. Implant loss as a result of peri-implantitis has been reported to range from 0 to 13.6% at the patient level and from 0 to 8.3% at the implant level [2]. The high prevalence of peri-implantitis ranging from 9.25 to 46.83% is a true challenge for the long-term success of dental implant surgery [3]. Plaque biofilm is the initiator of peri-implantitis; therefore, complete decontamination of the implant surface is the major target and first step of peri-implantitis therapy [4]. Several therapeutic interventions for plaque biofilm removal, including mechanical debridement, chemical disinfection, sustained release antibiotics, and regenerative/resective surgical therapy, have been used clinically; however, current therapies offer limited clinical improvements and have almost no microbiological improvements 6 months after treatment [5].

Erbium-doped yttrium–aluminum–garnet (Er:YAG) laser irradiation has been suggested to be a better treatment option for implant surface decontamination to control peri-implantitis because of its numerous advantages, including dental calculus removal, high bactericidal activity, excellent tissue ablation, and promoting of new bone formation [6]. Er:YAG laser has been extensively investigated for applications in clinical treatment of peri-implantitis either independently or in combination with other techniques, showing favorable outcomes [7]. However, there is no consensus on the optimal Er:YAG laser irradiation parameters for debridement of microstructured surfaces of titanium implants, particularly at a high pulse repetition rate [8]. Previous studies have shown that the contaminants clearance efficacy of Er:YAG laser is between 59% (80 mJ/pulse, 5 Hz) and 99.94% (120 mJ/pulse, 10 Hz) in a dose-dependent manner [9]. However, high energy of Er:YAG laser irradiation should be avoided to prevent the additional chemical contamination and minimize mechanical/thermal damage to titanium surface microstructures [10]. It has been showed that high power density of Er:YAG laser with an energy setting of 60–500 mJ/pulse leads to cracks on sandblasted and acid-etched (SLA) and polished titanium surfaces [11], or to peel off part of the layer on the surface of hydroxyapatite-coated (HA) implants [12]. Such alterations in surface morphology and physical/chemical properties may have either positive or negative effects on the biocompatibility of titanium implants, which may finally affect the procedure of re-osseointegration [13].

Before applying Er:YAG laser irradiation protocol clinically, in vitro investigation is necessary to determine the optimal settings for decontamination. Most current in vitro studies of Er:YAG laser irradiation have been mainly based on the construction of microbial membrane models in vitro [14]. The oral cavity is a complex environment, and the intraoral biofilm formation is particular and significantly different from the model constructed outside the mouth, which makes the research results distinct from clinic. Moreover, the limited studies on the biocompatibility of implant surfaces after Er:YAG laser decontamination are also mainly based on in vitro biofilm construction, and no consistent conclusions have been obtained [15, 16].

In this study, based on supragingival plaque biofilm construction on SLA and HA titanium surfaces in vivo, the decontamination efficacy of Er:YAG laser with different energy settings and their effects on the biocompatibility of titanium surfaces were investigated. The purpose of this study was to determine the appropriate energy of Er:YAG laser that can completely remove the biofilm without damaging implant surfaces and hampering biocompatibility. This study may provide a theoretical basis for the clinical application of Er:YAG lasers in the treatment of peri-implantitis.

## Methods

### Study subjects

Ten healthy volunteers (5 males and 5 females, age 24–27) were recruited as the study subjects. The study protocol was approved by the Medical Ethics Committee of West China Stomatological Hospital of Sichuan University (WCHSIRB-D-2018-060), and all participants signed informed consent forms. The inclusion criteria were as follows: (1) no history of systemic diseases or infectious diseases; (2) no history of radiotherapy and chemotherapy; (3) good oral hygiene (plaque index < 1); (4) no signs of chronic or destructive periodontitis or any inflammatory conditions of the surrounding soft tissues; (5) no use of antibiotics during the last three months; (6) nonsmokers. The exclusion criteria were as follows: (1) volunteer with defect of dentition; (2) inability to wear intraoral splint because of severe pharyngeal reflex; (3) bruxism.

**Fig. 1:**
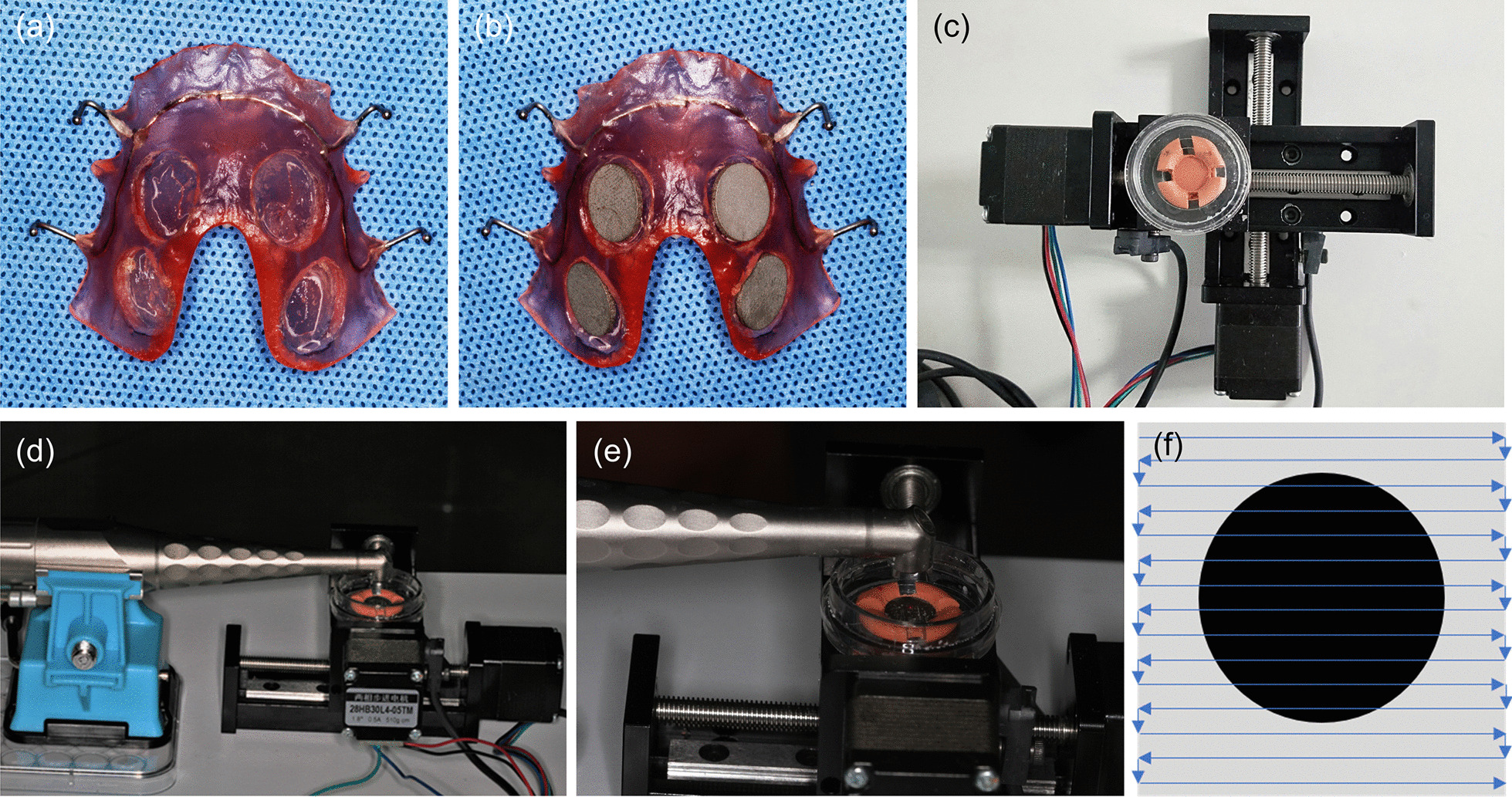
Plaque biofilm acquisition in vivo and Er:YAG laser decontamination procedure. **a** Acrylic splints. **b** Acrylic splints model carrying four titanium discs. **b** The mechanized and controllable experimental slide device. **b**–**e** Laser irradiation by using R02 handpiece on titanium specimens. **f** The scanning trace of laser decontamination

The upper jaw impressions of the volunteers were made to cast super-hard plaster models (Department of Implant, West China Hospital of Stomatology, Sichuan University). Then, acrylic splints with two circular holes with diameter of 10 mm on both the left and the right side were manufactured and sterilized with ethylene oxide gas (Chengdu Dental Technology Development Co., Ltd.); these splints would be retained in the mouth of the volunteers by the adjacent hook through adjacent space of the teeth (Fig. 1a). Then, the sterilized (103.4 kPa 121.3 °C, 30 min) SLA and HA titanium discs (commercially pure titanium grade II, 10 mm diameter, 2 mm thickness, Sichuan University National Engineering Research Center for Biomaterials) were attached in the circular holes of acrylic splints on the cleaning bench (Fig. 1b) and were placed in aseptic plastic bags and transferred to the volunteers.

### In vivo biofilm formation and scanning electron microscopy (SEM) observation

The participants wore the splints for 24, 36, 48 and 72 h without taken out of the oral cavity, allowing to brush their teeth only with toothbrush and maintain a regular diet. At the end of each period, the splints were taken off and rinsed twice with phosphate buffered saline (PBS); the titanium discs were removed from the splints and kept in 24-well plates in PBS. The titanium discs were fixed for 4 h with 2.5% glutaraldehyde at 4 °C, gently washed with PBS for 15 min, and dehydrated with a series of graded ethanol solutions (30%, 50%, 75%, 85%, 90%, 95%, and 100%), each concentration for 15 min. After drying at room temperature, the structure of the plaque biofilms on the specimen surfaces were examined using an SEM (Zeiss Leo 435 VP, Leo Electron Microscopy Ltd Cooperation Zeiss Leica, Cambridge, England) to determine the optimal wearing time for in vivo mature plaque biofilm formation. The contaminated titanium discs with an optimal biofilm formation period were chosen for the following assessment.

### Er:YAG laser irradiation procedure

SLA and HA titanium discs were divided into blank control (BC, clean discs) groups, experimental control (EC, contaminated discs) groups and experimental (EP, contaminated discs irradiated by Er:YAG laser) groups. EP groups were subjected to Er:YAG laser treatment (LightWalker ST-E, Fotona, Germany) using a mechanized and controllable experimental slide device (Fig. 1c). According to the instruction of manufacturer, for SLA surface specimen, the pulse duration and fluency erbium laser were set at 40 mJ/pulse at 10 Hz, 70 mJ/pulse at 10 Hz and 100 mJ/pulse at 10 Hz in MSP mode, respectively; and for HA coating surface specimen, the pulse duration and fluency erbium laser were set at 40 mJ/pulse at 10 Hz, 70 mJ/pulse at 10 Hz and 100 mJ/pulse at 10 Hz in SP mode, respectively. The titanium discs were fixed by the disposable sterile Petri dish in the center of the device, and the sample surfaces were irradiated by an Er:YAG laser using a R02 handpiece (Non-contact, 90°-angled dental handpiece with 0.6 mm spot size) with concomitant PBS flow cooling (5 mL/min), keeping the tip 1 cm away from the specimen surface perpendicularly (Fig. 1d, e). Parallel irradiation was conducted on each specimen by moving the working tip along a uniform scanning trace (Fig. 1f) with 1 mm scanning spacing at a constant speed of 7 mm/s for 1 min to cover the titanium surface. All prepared samples were stored in PBS solution.

### Characteristics of titanium surface after Er:YAG laser irradiation

All of the EC and EP groups were also examined using SEM to observe the surface pattern as well as the residual plaque biofilm. The contact angles of water on the surfaces of EC, BC and EP groups were also measured using the sessile drop method by standard type contact angle meter (Dropshapeanalyzer, DSA, KRSSGmbH, Germany). Double distilled water (4 μl) was deposited on the surface of all specimens. For each group, six individual specimens were tested to average the contact angle measurement.

### The efficacy of Er:YAG laser decontamination

Live/dead bacterial assay was used to observe the effect of laser decontamination. The EC and EP groups were analyzed using fluorescent detection by LIVE/DEAD BacLight bacterial viability Kits (Thermo Scientific, USA) using standard plate counts. The live bacteria were stained as fluorescent green (SYTO-9; excitation 488 nm), whereas dead bacteria were stained as fluorescent red (propidium iodide, PI; excitation 94 nm). All stained specimens were stored frozen at 4 °C, protected from light, then observed and captured by using laser scanning confocal microscope (LSCM, Olympus, Japan). Fluorescent plaque biofilm of randomly selected sites (×40 magnification) was imaged using NIS-Elements Viewer 4.2 software (Nikon, Japan).

Colony counting assay was used to quantitatively analyze the bacterial removal efficacy. The EC and EP groups were stored in 2 mL PBS and divided into six parallel groups. The microbial biofilm on the surfaces of the titanium discs was scraped from top to bottom with a transfer tip for 3 min each time and fully mixed with a vortex mixer (IKA, Germany) in 2-mL centrifuge tube. After serial tenfold dilution with aseptic PBS, aliquots of each dilution (100 µL) were spread on Brain Heart Infusion (BHI) agar plates. After anaerobically growing for 48 h in 80% N_2_ and 20% CO2 at 37 °C, for each plate, 50–300 colony-forming units (CFUs) were selected for colony counting. CFUs was determined as follows: CFUs/mL = the colony number in the whole plate × 10 × diluent times.

### Biocompatibility measurement after Er:YAG laser decontamination

For the biocompatibility measurement assay, all specimens of the BC, EC and EP groups were sterilized in a high-pressure steam turbine (103.4 kPa 121.3 °C, 30 min) and kept in a 48-well plate. Mouse calvarial osteoblastic cells (MC3T3-E1) were inoculated in cell culture flask in DMEM with 10% FBS and 1% penicillin/streptomycin at 37 °C under a humidified atmosphere of 95% air and 5% CO_2_. After the cultures reached 80–90% confluence, 0.5 mL of cell suspension of 2 × 10^5^ cells/mL was collected and seeded in each well of a 48-well plate containing different titanium specimens and incubated together under the same cell culture conditions. After 1 and 3 days of incubation, the cell cultured specimens were collected individually and fixed in 4% paraformaldehyde solution at 4 °C, gently rinsed with PBS for 15 min, and dehydrated with a series of graded ethanol solutions. After drying at room temperature, the adherence of cells on different titanium surfaces were observed by using SEM.

To quantitatively detect the effect of Er:YAG laser decontamination on different titanium surfaces, cell viability was measured using a cell counting kit (CCK-8, Dojindo Laboratories, Japan) following the manufacturer’s instructions. After 1, 2, 3, 5, and 7 days of cell culture, for each specimen in the 48-well plate, CCK-8 reagent was added into each well, and the plates were incubated at 37 °C for an additional 2 h. Four aliquots of 100 µL were transferred from each well into a new 96-well plate, and the optical density (OD) at 450 nm was measured using a microplate reader (Molecular Devices, Sunnyvale, USA).

### Statistical analysis

All data were subjected to statistical analysis using IBM SPSS version 21.0 software (IBM SPSS Statistics, v21.0; IBM Corp). The descriptive data were presented as the mean ± standard deviation (SD). All the experiments were performed in at least 3 independent experiments (n = 3) in triplicates. The normal distribution of data was analyzed by Kolmogorov–Smirnov test. Kruskal–Wallis test for non-normal distribution data and One-way ANOVA test for normal distribution data were used for statistical analysis to compare differences between the groups. The level of significance was set at *p* < 0.05.

## Results

### Plaque biofilm acquisition

**Fig. 2:**
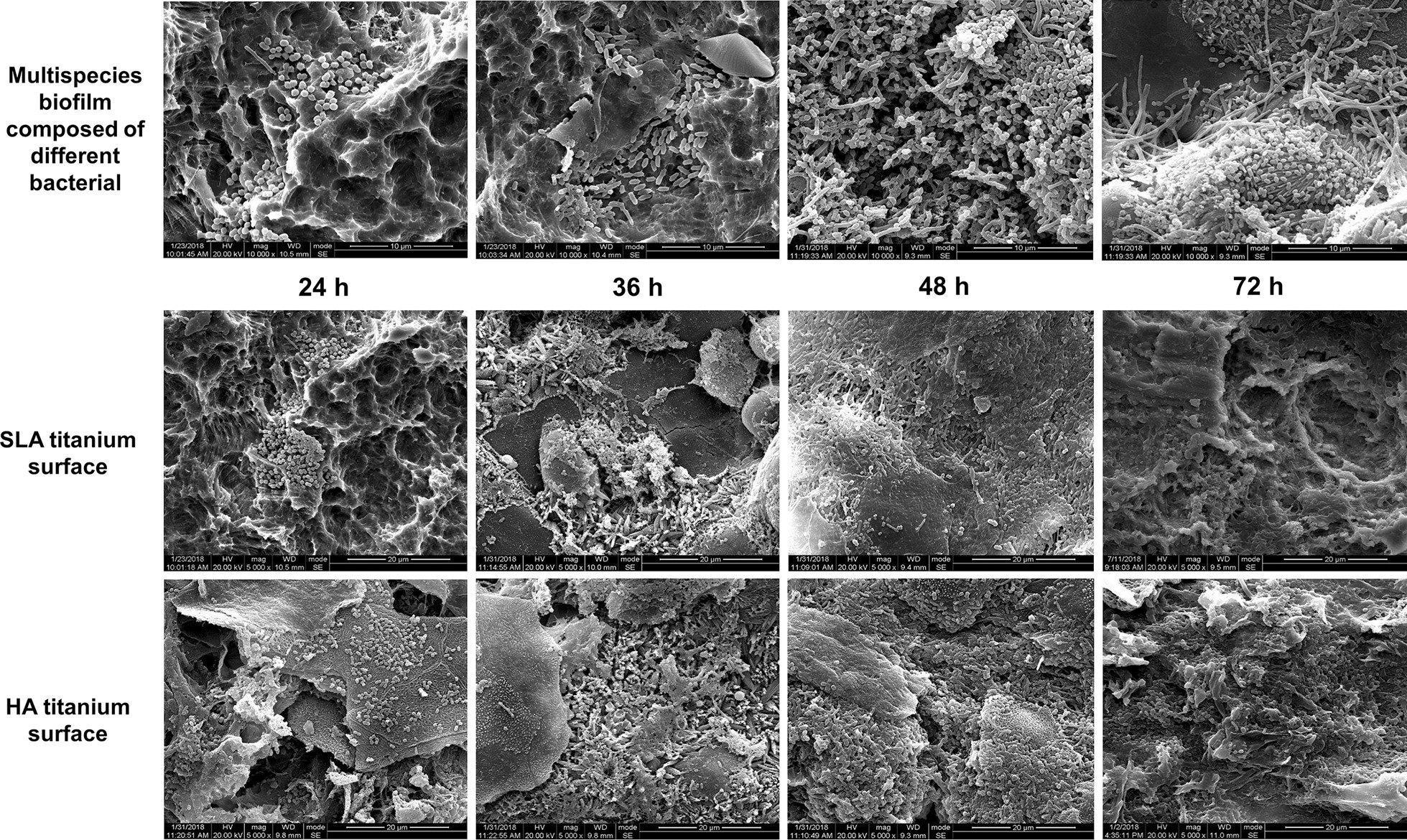
SEM images of SLA and HA titanium surfaces after establishment of biofilm within different wearing periods and different types of bacteria captured in the biofilm, including spherical coccus, rod-shaped bacillus, *Streptococcus mutans*, and a mixture of different bacteria

After plaque biofilm acquisition from volunteers in vivo, SEM images demonstrated that the SLA and HA titanium discs exhibited multispecies biofilm composed of coccoid-, rod-, and filament-shaped bacteria with occasional spirochetes which are arranged either as short streptococcal chains or as multicellular aggregates (Fig. 2). For both titanium discs, some bacterial aggregates appeared on the surface within 24 h; after 36 h, the early biofilm was formed but did not completely cover the discs. After 48 h, the titanium surfaces exhibited most concentrated bacterial cells completely covering the entire disc, accompanied with a large amount of extracellular matrix, indicating the mature biofilm formation. After 72 h, the matured biofilm began to fall off and the structure became loose (Fig. 2). Thus, 48 h was considered as the best wearing period of acrylic splints for volunteers to build up mature plaque biofilm.

### Morphology and wettability of different titanium surfaces after laser irradiation

**Fig. 3:**
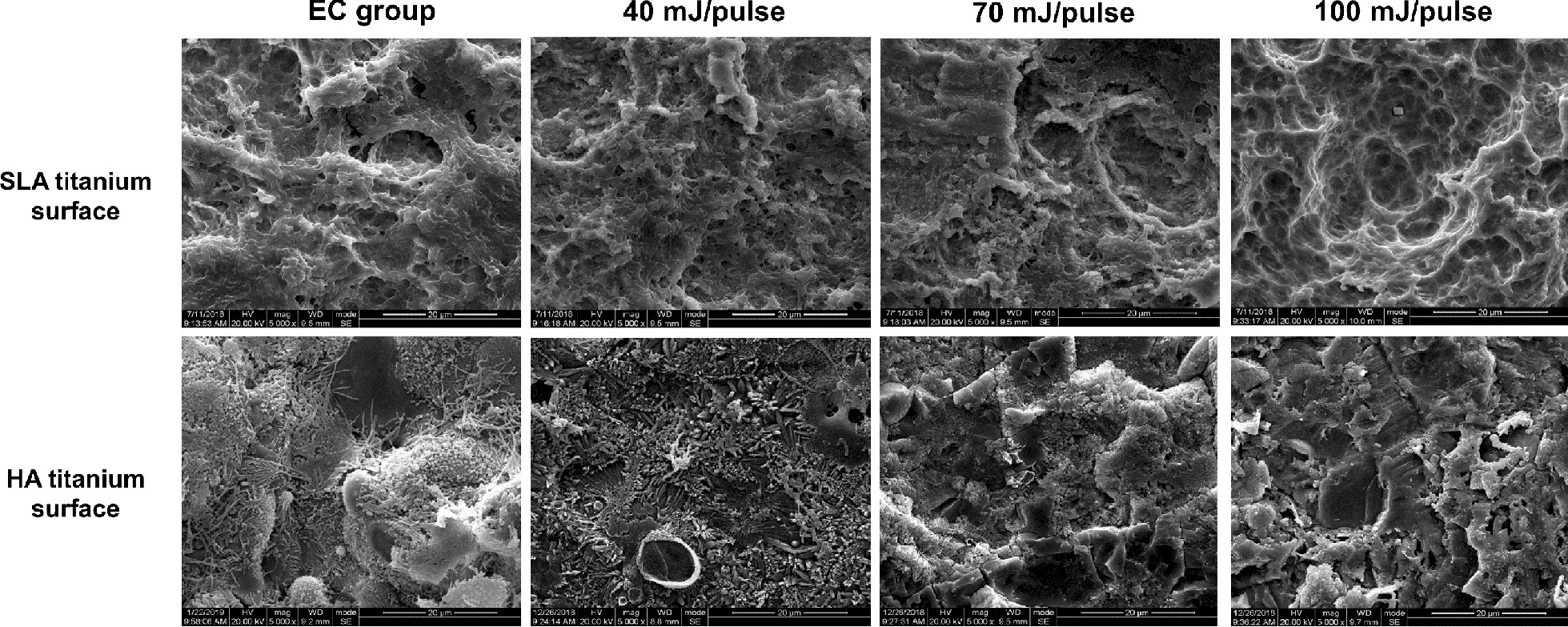
SEM images of the morphology of SLA and HA titanium surfaces before and after Er:YAG laser decontamination with different energy settings

The morphology of titanium surfaces before and after Er:YAG laser decontamination was observed under SEM. The results showed that the laser scavenging effect increased with the increasing of Er:YAG laser energy setting (Fig. 3). For SLA titanium discs, residual bacteria were still visible following irradiation exposure to 40 mJ/pulse, while complete biofilm was removed when the laser power was increased to 100 mJ/pulse. The surface structure demonstrated morphological changes following melt and ridges decreased or even disappeared, which was more obvious with a high laser energy setting. For HA titanium discs, almost no bacteria were visible, whereas remarkable surface melting was found on the surface when exposed to laser irradiation at 70 mJ/pulse; at higher energy of 100 mJ/pulse, the surface coating was almost completely peeled off or cracked, leaving the rough surface.

**Fig. 4:**
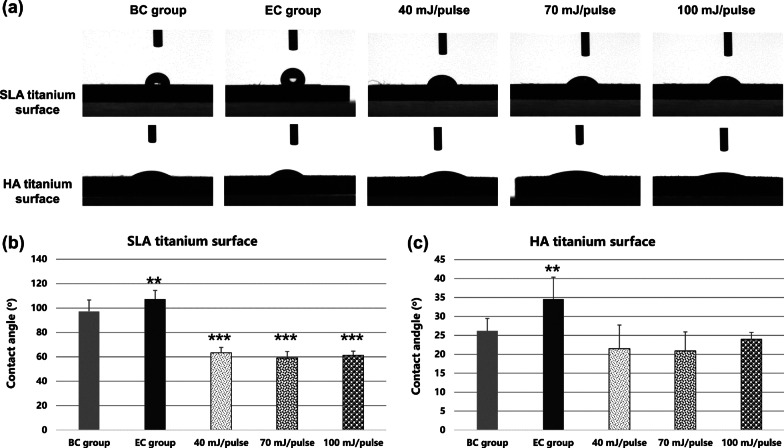
The contact angle of SLA and HA titanium surfaces before and after Er:YAG laser decontamination with different settings of energy. **a** Captured figures of water on different surfaces. **b** Contact angles of SLA titanium surfaces. **c** Contact angles of HA titanium surfaces. (BC = blank control group, EC = experimental control group) (**p < 0.01 and ***p < 0.001)

The contact angles of distilled water droplets on different titanium surfaces are shown in Fig. 4. In case of SLA titanium surfaces, the original contact angle was 97.12 ± 1.05° for the BC group and increased to 107.34 ± 7.05° for the EC group (*p* < 0.01). After Er:YAG laser irradiation, the contact angles significantly decreased to 63.32 ± 4.43°, 59.10 ± 1.28° and 61.27 ± 3.31° at laser energy settings of 40, 70, and 100 mJ/pulse respectively (*p* < 0.01), but there were no significant differences among different energy settings (*p* > 0.05). In the case of HA titanium surfaces, the original contact angle was 26.20 ± 5.78° for the BC group and increased to 34.61 ± 5.78° for the EC group (*p* < 0.01). After laser irradiation, the values decreased to 21.47 ± 6.26°, 20.88 ± 5.03°, and 23.98 ± 1.79° at energy settings of 40, 70, and 100 mJ/pulse, respectively (*p* < 0.01); however, there were no significant differences between the groups (*p* > 0.05) or compared with the BC group (*p* > 0.05).

### Decontamination efficacy of Er:YAG lasers irradiation

**Fig. 5:**
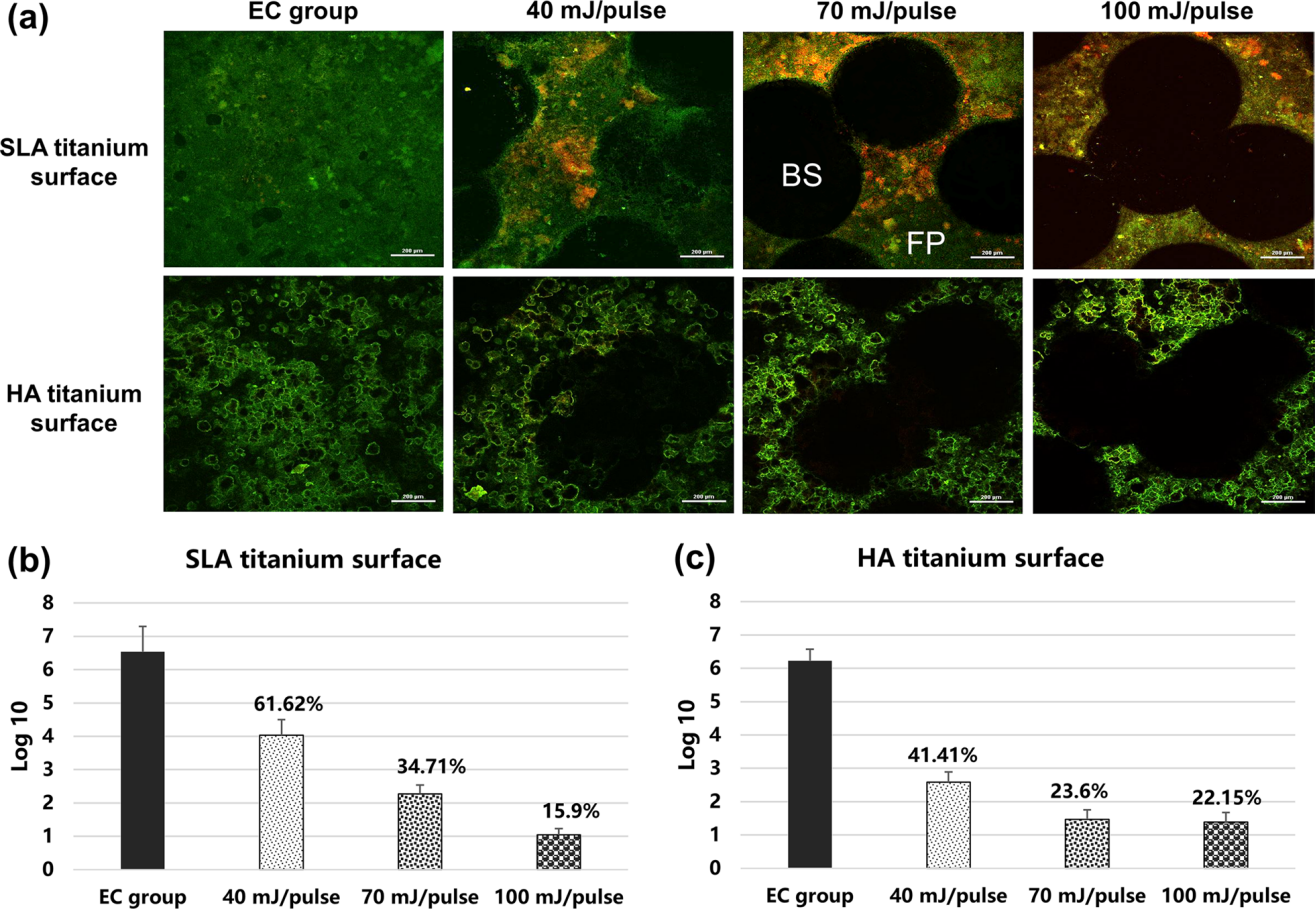
Decontamination efficacy of Er:YAG laser irradiation with different energy settings detected by live/dead bacterial fluorescent staining and colony counting assay against SLA and HA titanium surfaces. **a** CLSM photos of live/dead cells on titanium surface before and after laser decontamination; live bacteria were stained as green and dead bacteria stained as red. Black spots (BS) represent the irradiation spots and fluorescent parts (FP) area represents non-irradiation areas. **b**, **c** CFU counting results of SLA and HA titanium surfaces. The percentage of residual bacteria was calculated by the CFU ratio between the laser irradiation group and EC group. (EC = experimental control group)

Live/dead bacterial assay and colony counting assay were used to detect the decontamination efficacy of Er:YAG laser irradiation, and the results are shown in Fig. 5. In LSCM photos, the black spots (BS) are the irradiation areas and the fluorescent parts (FP) represent the residual biofilm without laser irradiation in which the live/dead bacterial are stained as green/red and the overlapping area is orange. For both the SLA and HA titanium groups, the area of decontaminated area did not expand with the increasing of Er:YAG laser energy, suggesting that Er:YAG laser irradiation cannot eliminate all biofilm on titanium surfaces. However, there was much larger orange area, including live and dead bacterial, at high energy settings for both the SLA and HA titanium groups, indicating that a higher energy of laser irradiation extended the range of disinfection potential around the irradiation spots.

Colony counting assay showed that CFU values of two different titanium surfaces significantly decreased with the increasing of laser energy settings (*p* < 0.01). For SLA discs, compared with the EC group, Er:YAG laser irradiation removed 38.38%, 63.29%, and 84.1% of bacteria from the original biofilm at energy settings of 40, 70, and 100 mJ/pulse, respectively. In the case of HA discs, Er:YAG laser irradiation removed 58.59%, 76.4%, and 77.85% of bacteria from the original biofilm at energy settings of 40, 70, and 100 mJ/pulse, respectively.

### Biocompatibility of different titanium surfaces after Er:YAG laser decontamination

**Fig. 6:**
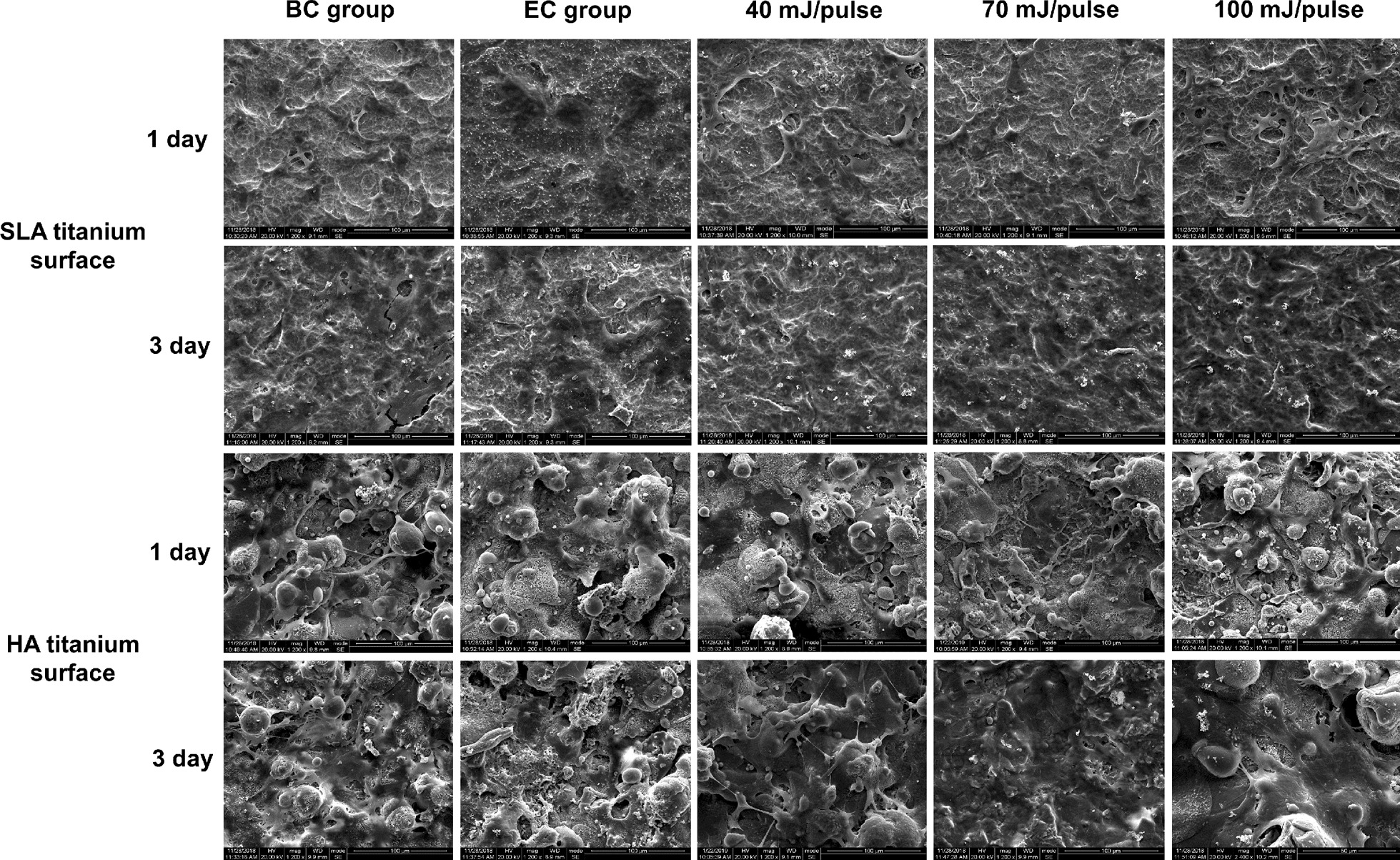
SEM observation of MC3T3-E1’s adhesion on SLA and HA surfaces before and after Er:YAG laser decontamination after 1 and 3 days of incubation. (BC = blank control group, EC = experimental control group)

The adhesion morphology of MC3T3-E1 cells cultured on SLA and HA titanium discs of each group (BC, EC, and EP groups) on day 1 and day 3 are shown in Fig. 6. SLA and HA titanium surfaces demonstrated similar modes of cell adherence. On the first day of incubation, the cells in the BC and EP groups began to attach to the surface in forms of spindle-shaped or irregular polygons without complete adherence, with the extending silk pseudopods distributed along the irregular surface of the material; the EC group showed less cell adhesion than the other groups. On the third day, the cells in the BC and EP groups proliferated significantly and formed completely flat adhesion on titanium surfaces. The cell number in the EC group also increased but was less than in the other groups, suggesting that biofilm contamination after high temperature and pressure disinfection still had potential interference on cell adhesion, and the negative effect was counteracted by Er:YAG laser decontamination.

**Fig. 7:**
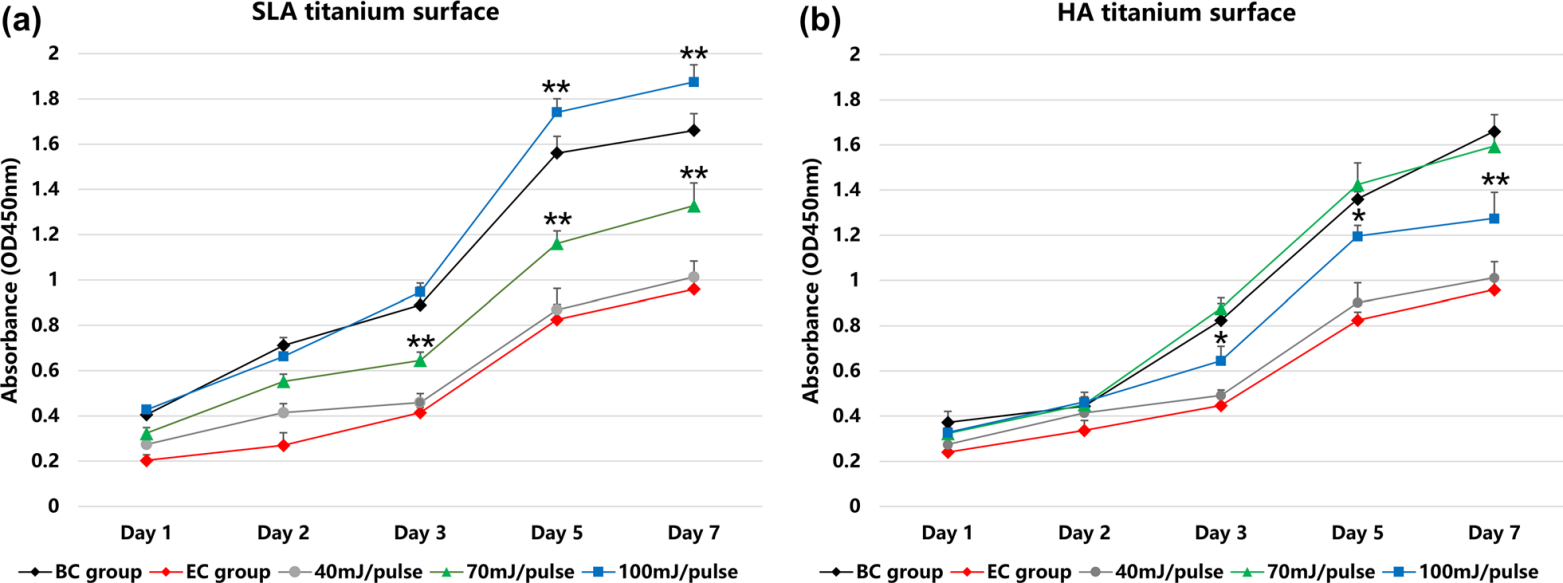
MC3T3-E1 cell proliferation curve on SLA and HA surfaces before and after Er:YAG laser decontamination. **a** SLA titanium surface. **b** HA titanium surface. (BC = blank control group, EC = experimental control group) (*p < 0.05 and **p < 0.01)

CCK-8 test was further conducted to quantitively detect the proliferation activity of MC3T3-E1 cells in different groups and the results are shown in Fig. 7. For SLA and HA titanium discs, the cell number in the EC group was significantly lower than that in the BC group in each period of incubation (*p* < 0.001), indicating that biofilm contamination negatively affected cell proliferation. After laser irradiation, the cell number increased significantly within the incubation time, except for the 40 mJ/pulse groups, for which most OD values were equal to those of the EC groups (*p* > 0.05). For SLA titanium discs, the OD values of the 70 mJ/pulse group were lower than those of the BC group; however, after 3 days of incubation, the OD values of the 100 mJ/pulse group exceeded the BC group, which lasted until the 7 days of incubation (*p* < 0.05 or 0.01). In the case of HA titanium discs, the OD values of the 100 mJ/pulse group were lower than those of the BC group; however, after 3 days of incubation the values of the 70 mJ/pulse group showed no statistical difference compared with the BC group (*p* > 0.05), indicating that Er:YAG laser decontamination affected the cell proliferation ability differently between SLA and HA titanium surfaces.

## Discussion

Considering that Er:YAG laser has been widely applied in clinical treatment of peri-implantitis and other related diseases, an increasing number of basic studies have focused on the efficacy of laser’s decontamination and its effect on titanium surfaces. However, most related studies of Er:YAG laser decontamination effects were based on extraoral single-bacterium models or multi-bacterium models [17]; there are few studies involving intraoral biofilm and none of them focused on the mature bacterial biofilm formation period in vivo [18]. Oral biofilms contain a complex ecosystem that harbors hundreds of bacteria; therefore, construction of single-bacterium biofilm or multi-bacterium biofilm in vitro may not be representative of a real in vivo situation. Generally, an intraoral acquired pellicle is formed after 2 h, in which bacterial population adheres to surfaces and allows the growth and reproduction of microbial communities as well as mature plaque biofilm formation within several days [19, 20]. Previous studies have shown that the early supragingival plaque could be obtained after volunteers wearing intraoral splints for 24 h [18, 21]. In this study, an early biofilm formed but it did not completely cover the titanium surface after 36 h; a mature biofilm was obtained after 48 h. The SEM micrographs demonstrated that the mature biofilm was mostly composed of coccoid-, rod-, and filament-shaped bacteria. The biofilm obtained in this study was an in vivo supragingival oral biofilm, which is different from subgingival plaque biofilms related to peri-implantitis. The biofilms in peri-implantitis form in anaerobic deep submucosal areas [22] and to date it is difficult to reproduce the same quality of submucosal biofilms in vivo or in vitro [23]; therefore, further studies are necessary to gain a better agreement between the actual circumstance and experimental results.

Er:YAG laser’s decontamination efficacy varies depending on irradiation parameter as well as on surface and specimen geometry. Er:YAG laser irradiations has been reported to be efficient in plaque biofilm removal over a wide range of powers, from 30 to 500 mJ/pulse [9, 10, 21, 24, 25]. Surface-specific modifications render a particular surface easier or more challenging to decontaminate. Quaranta et al. [26] reported that under the energy setting of 30 mJ/pulse at 10 Hz, Er:YAG laser showed different decontamination efficacy of 76.2%, 90.9% and 98.3% for mechanically treated, titanium slurry sprayed (TPS), and SLA titanium surfaces, respectively. Titanium surface geometry also affects the level of decontamination. According to Chen’s study, Er:YAG Laser cannot completely remove dental biofilms from rough titanium surfaces, and SEM images verified the presence of several bacteria in the valleys and undercuts of the rough surfaces of the implants [27]. In this study, Er:YAG laser with all energy settings (40, 70, and 100 mJ/pulse) effectively reduced the plaque on different titanium surfaces. Er:YAG laser’s decontamination efficacy varied with different titanium surfaces; at 100 mJ/pulse, laser irradiation was able to reduce about 84.1% and 77.85% bacterial counts on SLA and HA titanium surfaces, respectively. Live/dead fluorescence staining results demonstrated that even a high power of Er:YAG laser was not able to remove all bacterial biofilm. However, nearly complete or complete bacterial decontamination was obtained inside the irradiation spots. Moreover, many dead bacteria appeared outside of the irradiation area, and the numbers increased significantly with the increasing of laser energy settings, suggesting that the laser treatment not only removed the biofilm but also killed other bacteria on the titanium surface in a diffusion pattern. It has been reported that removing more than 96% of biofilms from the implant surface seems to be sufficient to achieve the peri-implant clinical health [28]. Thus, in clinical application, Er:YAG laser should be slowly moved to form overlapping areas of spot exposure to remove bacterial biofilms on contaminated implants as thoroughly as possible.

Er:YAG laser irradiation alters the characteristics of titanium surfaces, including the morphology, roughness and hydrophilicity [27]. Some studies showed no morphological changes on the surface of titanium implants treated with a suitable energy range of the Er:YAG laser [29, 30], whereas others reported opposite results [12, 24, 31]. Galli’s study [32] showed that Er:YAG laser at 200 mJ/pulse caused melting of SLA titanium surfaces; the thin crests of titanium were fused and collapsed into flat smooth plates. Shin’s study [24] indicated that Er:YAG laser with energies of 100 and 140 mJ/pulse caused no surface alterations; however, 180 mJ/pulse caused melting of SLA titanium surfaces. Shin’s study [24] also showed that for HA titanium implants, Er:YAG laser induced no significant surface alterations at 100 mJ/pulse, 10 Hz for 1 min; however, when the treatment time increased to 1.5 min and 2 min, the surfaces of HA titanium started to peel off. In addition, when laser intensities changed to 140 mJ/pulse, the surface of the implant became smooth due to the melting and cracks on the titanium surface. In this study, at low energies of 40 and 60 mJ/pulse, the SLA titanium surface structure demonstrated little morphological changes due to slight melting. When the energy increased to 100 mJ/pulse, the original sharpness of ridges completely disappeared, exposing the porous structure of the titanium discs. For HA titanium surfaces, the morphological changes were much different. HA coating began to melt at 40 mJ/pulse, started to peel off at 70 mJ/pulse and completely peeled off at 100 mJ/pulse, leaving the rough surfaces of titanium discs. This different phenomenon between SLA and HA titanium surfaces might be due to the infrared absorption peak of OH– in HA (2.8 μm) is similar to that the wavelength of Er:YAG laser (2.94 μm); thus a large amount of laser energy is absorbed by HA [33].

Hydrophilicity is an important element of implant design that affects biological responses, such as enhancement of the interaction between the implant surface and the biological environment [34] and the promotion of protein adsorption, as well as cell adhesion and diffusion [35]. Hydrophilicity is widely detected by the contact angle between material and distilled water; a smaller the contact angle reflects better hydrophilicity of the material. Er:YAG laser has been reported to reduce roughness and increase hydrophilicity of SLA titanium discs after irradiation. Specifically, Ayobian-Markazi’s study [15] demonstrated that original SLA titanium surfaces’ average contact angle was 133.4°, which decreased to 111.9° after Er:YAG laser irradiation at 100 mJ/pulse. In this study, the original contact angles of clean and contaminated SLA titanium surfaces were 97.12° and 107.34°, respectively; after Er:YAG laser decontamination, the contact angles decreased to 59.10°–63.32°, depending on the energy of laser treatment, but there was no significant difference among the groups of different intensities, indicating that Er:YAG laser decontamination significantly improved the hydrophilicity of SLA titanium surface. To date, few studies have focused on HA titanium hydrophilicity changes after laser irradiation. Our study was first to report that the contact angle of HA titanium surfaces was less than 30° without significant changes after irradiation at different energy settings, indicating that Er:YAG laser did not significantly change the hydrophilicity of HA titanium surface. Titanium surfaces with high surface energy and hydrophilicity can promote cell adhesion, proliferation and expression of markers related to cell differentiation and cell activity [17, 36, 37]. In this study, both SLA and HA titanium discs after laser irradiation were verified to be hydrophilic surfaces, which might have positive effects on the biocompatibility of titanium surfaces.

The interface reaction between titanium implants and the surrounding tissues plays an essential role in osseointegration, and biocompatibility of titanium surfaces is necessary for successful osseointegration [17]. However, there are scarce data on biocompatibility alteration of contaminated titanium surfaces after Er:YAG laser irradiation. Microbial biofilm and its residual cytotoxic products negatively affect the cellular behavior, including morphologic changes, proliferation and adhesion on titanium surfaces. It has been reported that attachment of gingival epithelial cells, gingival fibroblasts and osteoblast-like cells significantly decreased on biofilm-contaminated SLA titanium surfaces even after UV-light disinfection [17]. Er:YAG laser irradiation could not only remove the biofilm but could also change the physical and chemical characteristics of titanium surfaces, which might counteract the negative effect of the residual cytotoxic products from microbial biofilms [38]. Marco Giannelli’s study [16] stated that Er:YAG decontamination with 38.2 J/cm^2^ maintained a good biocompatibility of the pure titanium surfaces for Saos-2 osteoblasts proliferation. Sigrun Eick’s study [17] found that after Er:YAG decontamination, the high adhesion rate of osteoblast-like cells on titanium surfaces was even higher than that observed on pristine test specimens without any bacteria. In this study, SEM was used to observe the cell adhesion, and CCK-8 assay was used to detect the cell proliferation rate before and after laser treatment. SEM results suggested that Er:YAG laser decontamination might have potential to improve the adhesion of MC3T3-E1 on different titanium surfaces. Cell proliferation activity of the EC group was much lower than that of the BC groups for both SLA and HA titanium, confirming that the microbial biofilm disturbed the cell activities. For SLA titanium discs, after Er:YAG decontamination, the highest cell viability rate was observed in the 100 mJ/pulse laser group, and the values even exceeded those of the EC group after 5 and 7 days of incubation (*p* < 0.01); these results are consistent with the findings from Sigrun Eick’s study [17]. In the case of HA titanium discs, the highest cell viability rate appeared in the 70 mJ/pulse group; however, the values were comparable to those of the EC group (*p* > 0.05). Both titanium surfaces demonstrated good biocompatibility after Er:YAG laser decontamination, which might be due to the effective disinfection potential of the Er:YAG laser at certain energy settings, as well as due to the significant improvement of hydrophilicity by laser irradiation.

## Conclusion

According to the results obtained in the present study, it can be concluded that: (i) for SLA titanium discs, Er:YAG laser at 100 mJ/pulse is the optimal energy setting to effectively remove about 84.1% bacteria, markedly increase the hydrophilicity of titanium surfaces, slightly change the surface morphology, and significantly improve the MC3T3-E1’s cell adherence and proliferation activity compared with the BC group (clean titanium disc); (ii) for HA titanium discs, Er:YAG at 70 mJ/pulse is the most suitable energy setting, which effectively removes about 76.4% bacteria, has no effect on the hydrophilicity of titanium surfaces, demonstrates acceptable surface morphology alteration, and exhibits MC3T3-E1’s cell adhesion and proliferation activity similar to the BC group. The results of this investigation might provide useful information for suitable energy setting selection when applying Er:YAG laser irradiation to different titanium implants for clinical peri-implantitis therapy.

## Data Availability

All data generated or analyzed during this study are included in this published article.

## Abbreviations

ErYAG: Erbium yttrium–aluminum–garnet
SLA: Sandblasted/acid-etched
HA: Hydroxyapatite
BC: Blank control
EC: Experimental control
EP: Experimental groups
SEM: Scanning electron microscopy
LSCM: Laser scanning confocal microscope
BHI: Brain Heart Infusion
CFUs: Colony-forming units
MC3T3-E1: Mouse calvarial osteoblastic cells
CCK-8: Cell counting kit
OD: Optical density
SD: Standard deviation
BS: Black spots (BS)
FP: Fluorescent parts
TPS: Titanium slurry sprayed
